# A Narrative Literature Review on the Role of Exercise Training in Managing Type 1 and Type 2 Diabetes Mellitus

**DOI:** 10.3390/healthcare11222947

**Published:** 2023-11-11

**Authors:** Alessandro Piras, Milena Raffi

**Affiliations:** 1 Department of Life Quality Studies, University of Bologna, 40126 Bologna, Italy; 2 Department of Biomedical and Neuromotor Sciences, University of Bologna, 40126 Bologna, Italy; milena.raffi@unibo.it

**Keywords:** resistance training, aerobic training, physical exercise, exercise guidelines, glucose, insulin, glycemic control

## Abstract

Diabetes mellitus (DM) is a metabolic disease characterized by chronic hyperglycemia associated with impaired carbohydrate, lipid, and protein metabolism, with concomitant absence of insulin secretion or reduced sensitivity to its metabolic effects. Patients with diabetes mellitus have a 30% more risk of developing heart failure and cardiovascular disease compared to healthy people. Heart and cardiovascular problems are the first cause of death worldwide and the main complications which lead to high healthcare costs. Such complications can be delayed or avoided by taking prescribed medications in conjunction with a healthy lifestyle (i.e., diet and physical activity). The American College of Sports Medicine and the American Diabetes Association recommend that diabetic people reduce total sedentary time by incorporating physical activity into their weekly routine. This narrative literature review aims to summarize and present the main guidelines, pre-exercise cardiovascular screening recommendations, and considerations for patients with diabetes and comorbidities who are planning to participate in physical activity programs.

## 1. Introduction

Physical activity (PA) is normally suggested in the management of type 1 (T1DM) and type 2 (T2DM) diabetes mellitus and can improve glucose uptake by increasing insulin sensitivity, glucose transportation into the cells, and lowering body adiposity. In T2DM, the practice of PA, both alone and when combined with diet and drug therapy (e.g., oral hypoglycemic agents), can result in improved glycemic control [1]. In addition, PA can also help to prevent the onset of T2DM and has an important role in reducing the significant worldwide problem of this pathology. Different studies have enhanced our understanding of the acute and long-term physiological benefits of PA, although the precise duration, intensity, and type have yet to be fully elucidated [1,2,3]. In T1DM, however, the expected improvements in glycemic control with PA have not been clearly established. Instead, significant physical and psychological benefits of physical exercise can be achieved while careful education, screening, and planning allow the metabolic, microvascular, and macrovascular risks to be predicted and diminished [4].

The probable hypoglycemic advantage associated with PA was initially observed by Aristotle and later promoted by Allen and Joslin in 1920 [2]. Even though PA is considered one of the main solutions for diabetes management, indications about the exercise protocols for diabetes are scarce. Notwithstanding, different results have highlighted that many characteristics of the pathology can be improved and probably avoided following regular PA [2,3,4]. At the moment, we do not have recommendations regarding screening, exercise protocols, or treatment programs for people with diabetes. It is the same situation for personal frustration and support regarding insulin dosage, nutrition, and the potential limits on performance and safety [5].

It has been estimated that more than 600 million individuals will suffer from T2DM by 2035 [6]. Increasing attention has been paid to people at increased risk of developing T2DM, such as those living with impaired fasting glucose (i.e., prediabetes) [7]. PA is beneficial for preventing the progression of impaired fasting glucose to diabetes [8,9], but people at high risk of developing diabetes often fail to meet the PA guidelines of 150 min of moderate-to-vigorous aerobic exercise per week [10]. Innovative trials, such as the Diabetes Prevention Program (DPP), have demonstrated that a lifestyle intervention that comprises 150 min of moderate-intensity PA per week can reduce the progression of impaired fasting glucose by up to 58% compared to a non-exercise group and is almost twice as effective as the leading pharmaceutical intervention [11]. This represents a valid procedure designed to assess the potency of lifestyle modification for the prevention of T2DM [7]. Nowadays, even though the benefits of PA as a therapeutic measure for diabetic patients are well known and accepted, it is difficult to put exercise recommendations into action for several reasons. Insufficient knowledge among diabetologists and exercise professionals and a lack of dedicated facilities are indicated as important limitations [2]. Moreover, to provide effective PA support to people with diabetes, research is required to develop clinically effective, cost-effective, scalable interventions that bridge the gap between supervised PA advice. Print-based materials [12] and technology-based support, such as websites [13,14,15], smartphone apps [16,17], and telephone helplines [12,18], have also been shown to be effective in supporting people with diabetes to increase their level of PA. However, such interventions would have better efficacy if grounded in health behavior change theory [15]. For this reason, wearable technologies, offering biometric data to patients and healthcare professionals, bridging the gap between supervised PA advice, and enabling patients to engage in regular, long-term, physically active lifestyles [19,20], seem to be very helpful in diabetes prevention and management. It has been shown that professional exergames, as well as mobile app-based programs [21], have the potential to be effective treatment options, especially because they seem to at least partly solve the adherence problem [22]. Prescribing physical exercise is not generally undertaken, either by the general practitioner or by the diabetologist. This may be because there is insufficient awareness of the benefits of physical exercise or because there is a lack of specific knowledge regarding current recommendations. Thus, prescriptions, when suggested, are generic and more oriented towards ‘physical activity’ rather than ‘exercise therapy’, without appropriate indication about type, intensity, frequency, timing, progression, and precautions [2]. Furthermore, barriers to, and discriminations in, PA and exercise adoption and maintenance must be addressed to maximize participation [3].

This narrative literature review aims to highlight the literature available while examining the underlying physiology of exercise in diabetes, the benefits and risks of physical exercise, the strategies for minimizing complications, the protocols suggested, and the potential limitations.

## 2. Acute Effect of Exercise on Sugar Metabolism

PA allows for improving the glucose-tolerance curve by ameliorating insulin sensitivity in any subject, either with T2DM or T1DM [23]. Traditionally, physical exercise is promoted in T2DM, where insulin action is scarce in the context of insulin resistance and/or inappropriate insulin secretion. Nevertheless, even in the dysregulation of immune system function found in T1DM, the β-cell toxicity is facilitated by a complex interaction between oxidative stress and inflammation, for which the chronic exercise effect could be protective. During physical exercise, large changes in energy utilization require fine adjustments of glucose and fatty acid concentrations present in the blood. During the first 5–10 min of moderate-intensity exercise, a mixture of fatty acid and glucose provides the major fuel source for skeletal muscle. As exercise duration is prolonged and maintained at a moderate intensity, the contribution of fatty acid becomes more significant, thanks to hormonal responses, such as increased levels of norepinephrine and glucagon that promote the release of fatty acids from adipose tissue (fat stores) into the bloodstream (lipolysis) [24]. The liberated fatty acids circulate in the blood and are available for uptake by muscle cells. There is also an upregulation of fatty acid transporters in muscle cells. This increased expression of transport proteins facilitates the uptake of fatty acids from the bloodstream into the muscle fibers, making them available for energy production. The oxidation of fatty acids produces a larger amount of ATP compared to glucose metabolism. By relying more on fatty acids for energy, the body can conserve its limited glycogen stores [25]. Preserving glycogen is crucial for sustaining exercise performance, especially during endurance activities or when exercise intensity increases. Glycogen is the primary fuel source for high-intensity exercise, and by sparing its usage, it helps delay the onset of fatigue. There is a direct relationship between exercise intensity and glucose utilization: when the exercise intensity increases, we observe an augmented glucose utilization via the glycogen deposit from muscles and liver [25]. A complex hormonal and autonomic response allows an intensification in hepatic glucose production and tissue uptake by the mobilization of non-esterified fatty acid from adipose tissue deposits [24]. This is produced both by a fall in circulating insulin concentrations and a wide variety of counterregulatory hormones (glucagon, adrenaline, cortisol, and growth hormone) that counteract the hypoglycemic action of insulin [26]. Elevations in the blood concentrations of these hormones promote both increased glucose production and mobilization of non-esterified fatty acids from adipose storage sites. In addition, the production of new glucose in the liver (gluconeogenesis) from substrates such as lactate is enhanced [27]. Direct sympathetic stimulation of the pancreas and liver after muscle contraction may also bypass initial hormonal control and additional fuel supplies are provided by ketone formation and mobilization of lactate from inactive muscle glycogen. Glucose transport into muscle is again provided by the transporter protein GLUT4, which is recruited to the membrane surface in large quantities in contracting muscle, independently of insulin. Altogether, these changes maintain the increased fuel supply for exercising muscles and prevent hypoglycemia from excessive utilization [27].

Higher-intensity exercise or shorter-duration activities primarily rely on glycogen as the predominant fuel source, while lower-intensity or longer-duration exercise shifts towards a greater reliance on fatty acids for energy [25]. When exercise is ended, the body goes into a fasted state in which glycogen stores in muscle and liver are low and hepatic glucose production is enhanced. The level of the counter-regulatory hormones could remain higher for a long time with an associated hyperglycemic and hyperinsulinaemic response [28]. Improved GLUT4 transport and insulin sensitivity produce augmented glycogen resynthesis at the muscle level. Thereafter, when glycogen, glucose, and hormone levels return to normal levels, and homoeostasis is reached, insulin can produce supplementary glucose uptake and glycogen resynthesis in muscle and liver. When insulin is scarce, or the body is in a resistant state, glucose storage could be reduced within muscle as a consequence of the inadequate transport with an associated decrement in glycogen synthase activity [5,26].

## 3. Exercise Benefits for People with Diabetes

PA, sports, and exercise should be encouraged in people with T1DM for the same reasons as it should be promoted in people with T2DM or in the general population. The prescription of physical exercise for diabetic control should be considered for a variety of associated and independent health benefits. The full scope of these benefits can be seen in a number of reviews and include weight loss, weight loss maintenance, lipid profiles, reduction in blood pressure, good psychological profile, and the regulation of symptoms implicated in the metabolic syndrome [1,29]. It would appear that the combined effect of PA and diet provides the first and possibly most effective intervention in improving cardiovascular risk [30].

It has become evident that the most important effect of physical exercise is the improvement of blood sugar control, weight loss, and weight loss maintenance. PA helps in lowering blood sugar levels by increasing the uptake of glucose into muscles, even without the need for insulin. It enhances insulin sensitivity, making the body more efficient at using insulin to regulate blood sugar. Regular exercise can contribute to better long-term glycemic control. Moreover, physical exercise plays a crucial role in weight management, which is particularly important for individuals with T2DM. Regular PA reduces body fat, increases muscle mass, and improves overall body composition. Maintaining a healthy weight can enhance insulin sensitivity, allowing for more efficient glucose utilization. This can lead to decreased insulin resistance, which is a key factor in T2DM. By improving insulin sensitivity, physical exercise can reduce the reliance on medication or insulin therapy in managing diabetes [5,31,32].

PA can also improve cardiovascular health by strengthening the heart, reducing blood pressure, lowering LDL cholesterol levels, increasing HDL cholesterol levels, and improving blood circulation, significantly reducing the risk of heart disease and stroke. Cardiovascular complications are also worsened by stress, and PA is an excellent stress reliever [33]. Managing stress is important for individuals with diabetes because stress hormones can raise blood sugar levels. Physical exercise reduces stress levels, improves mood, and promotes overall mental well-being. By improving blood sugar control, weight management, cardiovascular health, and overall well-being, regular exercise can lower the risk of diabetes-related complications such as heart disease, stroke, nerve damage, kidney disease, and eye problems [5,31,32,33,34].

## 4. Exercise and Diabetes: General Recommendations

All levels of PA, including leisure activities, recreational sports, and competitive professional performance, can be performed by people with DM who do not have complications and are in good blood glucose control. A recent systematic review and meta-analysis of eight randomized controlled trials (5190 participants) reported that effective exercise interventions have the potential to reduce the personal and economic costs associated with diabetes [12,35]. They questioned the efficacy and cost-effectiveness of exercise referral schemes, increasing the exercise level and recommending further trials, especially those that are theory-driven [36]. It should be noted that many of the referral schemes used leisure centers for group-based programs, which did not provide expert supervision. James et al. [18] demonstrated a significant increase in PA among insufficiently active primary care patients who were provided a referral to receive five sessions from an exercise specialist. It has been suggested that those who potentially have the most to gain from exercise are often the ones who find it most difficult to engage in healthy behavior [35,37]. The effectiveness of PA as a therapeutic intervention is also seriously hampered by uncertainty concerning how clinical care teams should support people with diabetes to meet and maintain exercise guidelines [38].

It has been largely confirmed that daily PA has different implications in improving people's lifestyles by improving insulin sensitivity, reducing exogenous insulin injection, managing body weight and lipid profiles, boosting self-confidence, improving psychological problems associated with the pathology, reducing systemic inflammation, and most importantly, enhancing long-term protection against cardiovascular disease. Chronic hyperglycemia is supposed to be the mechanism by which coronary artery disease, stroke, nephropathy, retinopathy, and neuropathy occur over decades after disease onset. Moreover, hyperglycemia is related to increased inflammation, as reflected by activation of immune cells and increased systemic concentrations of proinflammatory cytokines/chemokines [39]. This inflammatory status persists for several hours or days after hyperglycemia has been resolved [40]. Preventing hyperglycemia is the main aim of DM treatment and may eliminate the excess inflammation caused by physical exercise. When this occurs, the subject can (i) postpone or cancel the planned exercise activity until the pro-inflammatory effects are reduced, (ii) perform physical exercise in a pro-inflammatory status, or (iii) reduce physical exercise-associated inflammation via medical treatment. Given that prior hyperglycemia alone (without ketosis) is not currently included in the contraindications to physical exercise, the second option is good [41]. General guidelines that may prove helpful in regulating the glycemic response to PA are presented below [2,42].

## 5. Metabolic Control before Exercise

I. Avoid physical exercise if the fasting glucose level is >250 mg/dL and ketosis is present, and take care if the glucose level is >300 mg/dL and no ketosis is present.
II. Ingest additional carbohydrates if glucose levels are <100 mg/dL.

Blood glucose monitoring before, during, and after physical exercise

I. Identify when changes in medication (insulin/hypoglycemic agent) or food intake are necessary.
II. Learn the glycemic response to different PA conditions.

### 5.1. Food Intake the Physical Exercise Day

I. Consume additional carbohydrates as required to avoid hypoglycemia.
II. Carbohydrate-based foods should be readily available during and after PA.

### 5.2. Food Intake the Rest Day

I. Reduce carbohydrate intake (<50 g/day).
II. A low carbohydrate diet has been shown to be effective for weight loss due to its effect on decreasing appetite and calorie intake.
III. Lowering dietary carbohydrate intake has demonstrated benefits for insulin resistance, the underlying cause of T2DM, by independently promoting both weight loss and a reduction in insulin levels.

## 6. Exercise in Managing T1DM

The ability to adjust the therapeutic regimen (insulin administration and timing, type and quantity of food ingestion before and after physical exercise) that allows safe participation and high performance has recently been recognized as an important management strategy in individuals with T1DM. In particular, the important role played by the patient in collecting self-monitored blood glucose data related to physical exercise responses to improve performance and enhance safety is now fully accepted [1,2,43].

When amateurs exercise at 50–60% of VO_2max_, which is below their anaerobic threshold, glucose levels rise following the increased uptake of skeletal muscle. The level of glycemia is generally reduced 30/45 min after PA, and it is preserved within the physiological range by two processes: a rapid increase in endogenous glucose production and a reduction in systemic insulin levels [41]. Patients should reduce or stop insulin infusion before exercise. This is to avoid the hypoglycemic effect due to the increase in glucose uptake at the skeletal muscle and to suppress endogenous glucose production.

Different is the situation in which the subject is involved in physical exercise above the anaerobic threshold and close to the VO_2max_. In this condition, the body produces a higher adrenergic activation that facilitates, beyond cardiovascular response, an endogenous increase in glucose level, exceeding the metabolic need at the peripheral level. This creates a state of moderate, transient hyperglycemia that does not exceed 140 mg/dL in healthy people. In T1DM, given that insulin cannot be secreted in response to this hyperglycemic response, the hyperglycemia value often continues to increase after exercise, sometimes becoming dangerous (>400 mg/dL) [41].

Hypoglycemia, which can occur during, immediately after, or many hours after PA, can be avoided. Indeed, during physical exercise, the body requires approximately 30–50% less insulin than in resting conditions to transport glucose across the cell membrane of myocytes. This is due to a considerable fraction of exercise-stimulated transmembrane glucose transport that occurs via noninsulin-dependent mechanisms [44]. In this case, it is important that the patient has both an adequate knowledge of the metabolic and hormonal responses to PA and well-tuned self-management skills. The increasing use of intensive insulin therapy has provided patients with the flexibility to make appropriate insulin dose adjustments for various activities. Moreover, in T1DM, after 2–4 years of disease onset, the ability to increase glucagon secretion (the main counterregulatory hormone) in response to hypoglycemia is permanently and completely abolished. However, in T1DM, hypoglycemia is caused by excessive exogenous insulin injection, involving low glycemia and high insulinemia that blocks glucagon release [41].

Aerobic performance is reduced in T1DM because of cardiovascular, muscular, and metabolic impairments. When compared to their nondiabetic counterparts, young patients with T1DM showed a reduction in VO_2max_ despite insulin therapy [45], a difference exacerbated in adults with neuropathic complications or sedentary lifestyles [46]. Different is the situation in which athletes with T1DM are compared to their nondiabetic counterparts, where VO_2peak_ was found to be similar [47]. Moreover, in T1DM, various cardiovascular parameters, such as end-diastolic volume and left ventricular ejection fraction, do not show a normal increment due to exercise, meaning that exercise can lead to normal aerobic and cardiovascular parameters [41].

The general recommendations for PA in adults with T1DM, free of complications, are the same for children, considering that kids are more subjected to a greater variability in glycemic level. For this reason, attention should be focused on glycemic fluctuation during exercise so that parents, teachers, and athletic coaches are properly trained. When dealing with adolescents, hormonal variations can increase the difficulty in managing glycemic levels. Notwithstanding these additional difficulties, it is out of the question that following the useful recommendations to avoid hypoglycemia, PA is a safe and satisfying practice for most children and adolescents with T1DM [42].

## 7. Exercise in Managing T2DM

A standard recommendation for people with and without diabetes is to start PA with a warm-up and end with a cool-down phase. The warm-up involves 5–10 min of aerobic exercises (walking, cycling, etc.) at low intensity. The aim is to prepare the skeletal muscles, heart, and lungs for a progressive increase in exercise intensity. Then, muscles can be gradually stretched for an additional 5–10 min to maintain a good range of motion in the joints [48]. After the main training session, a 5–10 min cool-down should be organized similarly to the warm-up. The aim of the cool-down is to gradually bring the heart rate back to its pre-exercise value. There are some recommendations that are particularly important and specific for people with T2DM. Moderate weight training exercises with light weights and high repetitions can be suggested for maintaining or enhancing upper body strength in people with diabetes. Different long-term studies have established long-lasting beneficial effects of regular PA on carbohydrate metabolism and insulin sensitivity, which can be maintained for at least 5 years. They suggested PA programs with an intensity in the range of 50–80% of VO_2max_, three to four times a week, for 30–60 min a session. Aerobic exercise should be suggested, considering safety measures for PA involving the feet, which are essential for many patients with diabetes [1,2,48,49,50,51]. High-resistance exercise using weights may be acceptable for young individuals with diabetes but not for older individuals with long-standing diabetes.

Good hydration is also crucial, as dehydration can adversely affect glycemic levels and heart function. Through PA, fluid should be frequently consumed to compensate for losses in sweat reflected in body weight loss or the maximal amount of fluid tolerated (e.g., 0.5 L of fluid consumed 2 h before PA) [52]. Precautions should be taken when exercising in extremely hot or cold environments.

It has been shown that improvements in glycated hemoglobin (HbA1c) are generally 10–20% of the baseline and are most marked in patients with mild T2DM and in those who are likely to show insulin resistance [53]. It remains true, unfortunately, that most of these studies suffer from inadequate randomization and controls and are confounded by associated lifestyle changes. The general agreement is that regular exercise should not be expected to dramatically affect HbA1c values; other variables, such as increased food intake or reduced insulin dosages, compensate for any increases in glucose disposal. Nonetheless, epidemiological evidence confirmed that being physically active, rather than sedentary, can lower mortality and morbidity for any given level of HbA1c [54].

It seems that long-lasting programs of PA are good enough and demonstrate a higher rate of adherence in patients with prediabetes or uncomplicated T2DM. In this kind of study, researchers started their training programs with initial supervision, followed by home-training programs with follow-up to assess the level of adherence [4]. A lot of them have shown good results in the maximum oxygen consumption with few complications for patients [55].

## 8. Psychological Profile, Muscular, and Cardiovascular Evaluations

Other important aspects to consider when prescribing an exercise program are the evaluation of motor responses and the consideration of the psychological profile. Evaluations represent a fundamental moment for understanding the real capabilities of an individual, setting up protocols and assessing results over time. People with diabetes show both impaired exercise tolerance and an excessive risk of developing heart failure, which are not entirely explained by known cardiovascular risk factors or coronary artery disease [56]. The risk for cardiovascular disease and other diabetes-related complications, including neuropathy, retinopathy, and nephropathy in persons with long-standing disease, is high, and care should be taken to properly screen individuals before recommending a new exercise program. Caution is warranted for those with advanced disease complications and medical screening; before initiating any new vigorous exercise program, a graded exercise stress test with ECG and blood pressure monitoring should be performed. The main assessments that should be made before carrying out any type of physical exercise are, in order: (i) the Health Assessment Questionnaire; (ii) assessment of balance level and risk of falls; (iii) cardiopulmonary exercise testing; (iv) the assessment of muscle strength.

Health assessment questionnaires [57]. While several tools are available to measure health-related quality of life (HRQoL) for patients with diabetes, the design and, therefore, duration of these measurements may limit their feasibility in the daily routine of a sports facility. Furthermore, these measures do not distinguish items for diabetes-specific quality of life. Thus, a specific questionnaire on the diabetics’ quality of life (DMQoL) was developed with only 10 questions, sensitive to the change related to the progression of diabetes compared to the initial stages (e.g., glycemic changes). The combination of the DMQoL and the WHOQOL-BREF (the shortened version of the quality of life questionnaire designed by WHO) provides a comprehensive picture of overall health-related quality of life in patients with diabetes and improves the ability to detect changes clinically significant to the pathology.

Tests that determine the level of balance and the risk of falls. Patients with T2DM, particularly those ≥65 years old, exhibit an increased rate of falls. It is, therefore, important to assess the risk of falling before the prescription of an exercise intervention. The most used tests are the Timed Up and Go Test (TUG), the Functional Reach Test, (FRT), the Berg Balance Scale (BBS), and the Dynamic Gait Index (DGI). Among those tests, the TUG showed the greatest sensitivity (90%) and specificity (88%) to the phenomenon [58]. In this assessment, the patient gets up from a chair, walks 3 m, turns around, returns to the chair, and sits down again. This task must be completed within 10.6 s. Times between 11 and 20 s are within the normal range for frail elderly and disabled patients; times ≥20 s indicate that the person needs external assistance. A score ≥30 s predicts a higher risk of falling.

Cardiopulmonary exercise testing (CPET). Measurements of ventilation, gas exchange, and electrocardiography during an incremental exercise test are noninvasive protocols that provide an assessment of pulmonary, cardiovascular, and muscle function during exercise (see Table 1). The addition of echocardiographic monitoring (“imaging-CPET”), mainly used in patients with heart failure, may provide further insight into different aspects of cardiac function during exercise and their impact on exercise intolerance [59]. Even though guidelines recommend the application of objective exercise prescriptions using CPET data [59], it is common to find programs without CPET information or with limited resources to establish exercise intensity on the basis of resting heart rate (e.g., exercising heart rate threshold set 20 or 30 beats/min above the resting heart rate). This simple method has been criticized and demonstrated to be inadequate by several researchers. Moreover, it should be noted that performing this test routinely and its cost mean that specific criteria must be defined regarding patients for whom it would be imperative to perform the test.

Assessment of muscle strength. A significant percentage of T2DM patients (prevalence 16%, mean age 58 years old) are sarcopenic compared to age-matched healthy individuals [34]. Considering that 80% of insulin-mediated glucose uptake occurs in skeletal muscle (lean mass) [60], we should consider the importance of increasing lean mass for improving glycemic control in these individuals [61]. Furthermore, muscle strength is reduced by 30% to 50% in T2DM patients compared to their healthy counterparts. Dynamometry is considered the gold standard for examining muscle strength. However, considering the cost of this device and the technical skills required, this test is not feasible for those working in private and home care settings. There are different types of tests, such as handgrip strength and sit-to-stand tests. Handgrip strength shows moderate to high correlations with extremity muscle strength. The patient sits in a chair with their elbow flexed to 90 degrees and a force device in one hand. Subsequently, the patient grips the device as tightly as possible for 3 s. This test is performed three times, alternating hands. Another strength test that could be considered to assess muscle strecngth in T2DM patients is the sit-to-stand test, although the validity of this test is currently under intense debate. Briefly, in this test, the person, without the help of the hands and arms, but with only the work of the legs, must perform, in 1 min, as many reps as possible and sit with legs bent at 90 degrees. Another version foresees a 30 s duration (30s-STS) [62], and another comprises five reps executed as quickly as possible (FTSST) [63].

## 9. Exercise Prescription

Regular exercise enhances overall fitness, strength, and endurance [61]. It boosts energy levels and can alleviate symptoms of fatigue commonly experienced by individuals with diabetes. It is important for individuals with diabetes to consult with their healthcare team before starting an exercise program. They can provide guidance on the type, intensity, duration, and frequency of exercise that suits individual needs and medical conditions. Additionally, monitoring blood sugar levels before, during, and after exercise is crucial to prevent hypoglycemia or hyperglycemia episodes.

As soon as the patient has been inspected and the risk factors and exercise tolerance identified, regular exercise should be suggested. Most patients with T2DM are sedentary, overweight, and middle-aged or older. In this population, PA may well be beneficial but needs to be carefully applied. Guidelines published by the American Diabetes Association (ADA) and by the American College of Sports Medicine (ACSM) suggest a proper warm-up of 5–10 min, followed by stretching, then the main activity session, ended with 5–10 min of an active cool-down epoch to bring back the physiological variables to their pre-exercise values [49]. The intensity, duration, and frequency of exercise necessary for good health should be in the range of 60–80% of maximal oxygen consumption delineated in the ACSM guidelines in 1976 [32]. The aim of an adult should be to maintain continuous moderate exercise for 30 min, equivalent to brisk walking, for five or six days a week, with the possibility to do shorter bouts of more intense exercises. Exercises at vigorous intensity are widely suggested for their health benefits and can be safely recommended for people with diabetes if cardiovascular and hypertensive complications are considered [5,32]. To our knowledge, no studies have accurately defined the most suitable exercise programs for people with diabetes up until now. It is unsuitable to be too prescriptive, and instead, we should concentrate on adherence and compliance. When ACSM guidelines are suggested, there is a dropout rate of 40–70% after 12–18 months, even with an active intervention program [48]. Nevertheless, recent guidelines have gained wider acceptance, and much greater success has been shown in the Malmo intervention studies with mixed high and low-intensity exercises, although exercises below 30% VO_2max_ have demonstrated lower benefit [64].

PA guidelines for patients with DM are the same as for healthy people unless co-occurring health conditions or advanced age affect their physical ability. In particular, interventions that combine aerobic and resistance training appear to show greater efficacy than the two training modalities taken individually [65]. Combined exercise three times/week may be of greater benefit for glycemic control than aerobic or resistance exercise alone. Additionally, total exercise duration and calorie expenditure were greater with the combo workout than individual workouts [65]. Several types of exercise protocols enhance health and glycemic management in individuals with DM, although structured exercise training has been studied most frequently, with benefits resulting from enhanced insulin sensitivity, reduced postprandial hyperglycemia, and reduced cardiovascular risk.

### 9.1. High-Intensity Interval Training (HIIT)

There has been a growing interest in high-intensity interval training (HIIT) over the past 20 years. HIIT has received considerable attention due to its positive metabolic and cardiovascular adaptations that are similar, or even superior, to moderate-intensity continuous training in a variety of populations [7,66,67]. Specific to diabetes prevention, a recent meta-analysis demonstrated that HIIT leads to greater improvements in insulin resistance compared with training at moderate intensity [68]. Given the positive health adaptations, HIIT may represent a promising PA strategy for individuals with impaired fasting glucose [7]. HIIT involves alternating short sets of high-intensity exercise, typically achieving values ≥85% peak heart rate (HRmax), with passive recovery periods or light exercise typically performed at ≤70% HRmax. HIIT has been proposed as a time-efficient form of exercise. A typical HIIT session can be up to three times shorter than that of traditional moderate-intensity continuous training (MICT) and can lead to both cardiovascular and metabolic improvements in less time than MICT [69,70]. Several authors have studied the potential benefits of HIIT in multiple aspects, including cardiorespiratory fitness, anthropometric variables, mental health, and cardiovascular and cardiometabolic diseases in different populations, both healthy and with pathologies [7,69,71]. In individuals with T2DM and without complications, training adaptations induced by HIIT and MICT are equally capable of rapidly attenuating some local limiting factors governing the initially impaired VO_2_ kinetics response during submaximal exercise [72,73]. For this reason, HIIT at low volume should also be considered a suitable and effective exercise modality to enhance oxidative metabolism in individuals living with T2DM [74]. However, there are limited data on people’s adherence to HIIT in the long term and outside of a supervised or laboratory environment. Preliminary evidence shows that individuals with impaired fasting glucose can independently adhere to HIIT training for more than 4 weeks, and they do so at a higher rate with respect to the adherence to moderate-intensity continuous training [69]. While these findings are encouraging, more research is required to determine if samples drawn from a pre- or diabetic population can adhere to long-term interval exercise [75].

### 9.2. Peripheral Heart Action Training

A particular form of HIIT is PHA (Peripheral Heart Action) training [76]. PHA is a circuit training exercise that involves several stations that involve different muscles or muscle groups, following a well-defined pattern: the alternation between the exercises that involve the muscles located “above and below” the heart in such a way as to avoid passive breaks between stations. The HIIT session is repeated five times in the first 2 days of training and gradually increased in subsequent sessions according to the subject’s heart rate, which is monitored with the heart rate monitor. In the PHA session the subjects perform 15 repetitions on each piece of equipment and then move on to the next station with active breaks until the completion of the circuit of training. Active breaks imply that the subjects train the lower limbs as soon as they finish the upper limbs and vice versa. This circuit training is performed four times, separated by 1 min of rest, and resistance is increased for the next exercise session if the subject is able to perform 15 full repetitions during the final set for each exercise. Subjects wear a heart rate monitor and maintain intensity at 55–60% of 1RM, which corresponds to approximately 60–80% of HRmax.

### 9.3. Water Exercise

Compared to land-based exercise, aquatic exercise displays different advantages, including its effects on cardiovascular regulation because the blood flow to the lower limbs decreases due to the existence of hydrostatic pressure. This increases the redistribution of blood flow and the cardiac preload, thus increasing the stroke volume. Moreover, due to the height of the water, the pressure on systemic circulation increases, further affecting respiratory effort. These changes are helpful in increasing the elasticity and strength of the respiratory muscles, improving oxygen uptake [77]. The resistance and heat dissipation effects of water are conducive to energy expenditure and improve the effects of exercise [77]. Therefore, exercise in water could be considered an alternative protocol to land-based exercise. Such protocol could be started with a 5 min warm-up consisting of articular mobilization, followed by 30 min of easy swimming (alternating the various swimming styles—front crawl, breaststroke, backstroke and butterfly), 5 min of leg-only swimming and 5 min of arm-only swimming, 5 min of aquatic skills, and 5 min of cool-down. An alternative protocol, with head-out water immersion, may be used successfully and induce positive metabolic adaptations in patients with diabetes [78].

Exercise prescription must also consider patients’ readiness to exercise, attitudes, and belief systems while positively encouraging decisions to exercise. Support can be provided through a team of doctors, nurses, physiotherapists, lifestyle counsellors, and exercise consultants and even through health policy decision-making at the government and local levels. Moreover, exercise should be prescribed based on the type of diabetes, distinguishing between type 1 and type 2.

## 10. Conclusions and Future Recommendations

The major challenge is to persuade diabetic people to practice PA and to follow dietary recommendations. Sedentary persons with disabilities are very resistant to changing their lifestyle; in particular, adult diabetic people are resistant to changing their habits, maybe because of this pathology, except for those who have complications. Successful interventions to promote long-term changes have used specific strategies to promote sustainable effects of interventions [79]. Considering that drug-resistant diseases could become the leading cause of death by 2050, an intervention aiming at sustainable lifestyle change must, therefore, include components that facilitate the maintenance of PA levels and dietary changes over time.

It is well known that men and women of all ages and abilities can improve their quality of life through regular PA associated with well-designed dietary recommendations and nutrition therapy. Being physically active is one of the most important actions that people of all ages can take to improve their health. The regular practice of physical exercise fosters normal growth and development, makes people feel better, function better, sleep better, and reduces the risk of a large number of chronic diseases. Health benefits start immediately after exercising, and even short episodes of PA are beneficial. Evidence regarding the health benefits of regular PA is well established, and research continues to provide insight into what works to get people moving, both at the individual and community levels. Achieving the benefits of PA depends on our personal efforts to increase activity in ourselves, family, friends, patients, and colleagues. Action is also required at the school, workplace, and community levels. Future recommendations for elderly interventions should emphasize the importance of naturalistic or personally meaningful environments and designs that should induce a mismatch of supply and demand; they should have high task variability, fulfilling basic individual senior needs, but also be engaging to maximize long-term adherence to physical exercise and an active lifestyle.

## Figures and Tables

**Table 1:** Physiological variables measured during CPET.

Physiological Mechanism	Direct Measurement	Indirect Measurement
General	VO_2_; peak power	VO_2_%
Cardiovascular system	HR; BP	VO_2_/HR
Cardiac electrical system	ECG	Chronotropic insufficiency; change in heart rate of recovery
Ventilation	Ventilation; Tidal volume	VE/VO_2_; VE/VCO_2_
Gases exchange	SpO_2_; PETO_2_; PETCO_2_	Anaerobic threshold
Skeletal muscles	Scala di Borg	Δ(a − v)O_2_; Anaerobic threshold
Metabolism	RER	Anaerobic threshold
Systolic function	LVEF; TAPSE	Systolic reserve
Diastolic function	E/A; DT	Systolic reserve

Legend: VO_2_%, percentage of peak oxygen uptake; HR, heart rate; BP, blood pressure; ECG, electrocardiography; VE/VO_2_; VE/VCO_2_, ventilator equivalents of oxygen and carbon dioxide; SpO_2_, arterial oxygen saturation; PETO_2_ and PETCO_2_, end-expiratory pressure of oxygen and carbon dioxide; Δ(a − v)O_2_, arteriovenous difference in oxygen; RER, respiratory exchange ratio; LVEF, left ventricular ejection fraction; TAPSE, tricuspid annular plane systolic excursion; E/A, transmittal flow velocity; DT, deceleration time.

## Data Availability

Data are contained within the article.
